# Clinical Value of Information Entropy Compared with Deep Learning for Ultrasound Grading of Hepatic Steatosis

**DOI:** 10.3390/e22091006

**Published:** 2020-09-09

**Authors:** Jheng-Ru Chen, Yi-Ping Chao, Yu-Wei Tsai, Hsien-Jung Chan, Yung-Liang Wan, Dar-In Tai, Po-Hsiang Tsui

**Affiliations:** 1 Department of Medical Imaging and Radiological Sciences, College of Medicine, Chang Gung University, Taoyuan 333323, Taiwan; fierens601@gmail.com (J.-R.C.); liwes215@gmail.com (Y.-W.T.); jsz82513@gmail.com (H.-J.C.); ylw0518@gmail.com (Y.-L.W.); 2 Department of Computer Science and Information Engineering, College of Engineering, Chang Gung University, Taoyuan 333323, Taiwan; yiping@mail.cgu.edu.tw; 3 Graduate Institute of Biomedical Engineering, Chang Gung University, College of Engineering, Taoyuan 333323, Taiwan; 4 Department of Neurology, Chang Gung Memorial Hospital at Linkou, Taoyuan 333423, Taiwan; 5 Department of Medical Imaging and Intervention, Chang Gung Memorial Hospital at Linkou, Taoyuan 333423, Taiwan; 6 Institute for Radiological Research, Chang Gung University and Chang Gung Memorial Hospital at Linkou, Taoyuan 333423, Taiwan; 7 Department of Gastroenterology and Hepatology, Chang Gung Memorial Hospital at Linkou, Chang Gung University, Taoyuan 333423, Taiwan

**Keywords:** ultrasound imaging, information entropy, deep learning, hepatic steatosis, fatty liver

## Abstract

Entropy is a quantitative measure of signal uncertainty and has been widely applied to ultrasound tissue characterization. Ultrasound assessment of hepatic steatosis typically involves a backscattered statistical analysis of signals based on information entropy. Deep learning extracts features for classification without any physical assumptions or considerations in acoustics. In this study, we assessed clinical values of information entropy and deep learning in the grading of hepatic steatosis. A total of 205 participants underwent ultrasound examinations. The image raw data were used for Shannon entropy imaging and for training and testing by the pretrained VGG-16 model, which has been employed for medical data analysis. The entropy imaging and VGG-16 model predictions were compared with histological examinations. The diagnostic performances in grading hepatic steatosis were evaluated using receiver operating characteristic (ROC) curve analysis and the DeLong test. The areas under the ROC curves when using the VGG-16 model to grade mild, moderate, and severe hepatic steatosis were 0.71, 0.75, and 0.88, respectively; those for entropy imaging were 0.68, 0.85, and 0.9, respectively. Ultrasound entropy, which varies with fatty infiltration in the liver, outperformed VGG-16 in identifying participants with moderate or severe hepatic steatosis (*p* < 0.05). The results indicated that physics-based information entropy for backscattering statistics analysis can be recommended for ultrasound diagnosis of hepatic steatosis, providing not only improved performance in grading but also clinical interpretations of hepatic steatosis.

## 1. Introduction

Hepatic steatosis is a typical nonalcoholic fatty liver disease (NAFLD) that can progress to steatohepatitis, fibrosis, cirrhosis, and even hepatocellular carcinoma [1,2]. Early detection and treatment of hepatic steatosis is helpful in halting NAFLD [2]. Liver biopsy remains the gold standard for grading hepatic steatosis; however, it is inappropriate for screening and routine use because of its invasiveness and potential for complications.

Ultrasound grayscale imaging (B-mode) is the modality most commonly employed for screening NAFLD and grading hepatic steatosis because of its cost effectiveness, accessibility, and real-time capability. The assessment of hepatic steatosis is typically based on observations of the liver echotexture, echo penetration, visibility of the diaphragm, and clarity of liver vessel structures [3]. However, the subjective nature of B-mode ultrasound results in relatively poor interobserver agreement [4]. The accuracy of diagnosis also depends heavily on the experience of the operator. For these reasons, the use of quantitative ultrasound has been explored by researchers and has been shown to be of value in reducing diagnostic uncertainty.

Several quantitative ultrasound approaches have been investigated for their utility in the detection of hepatic steatosis in NAFLD. In general, these analysis methods can be designed based on either the texture descriptions of ultrasound images or the information extraction of echo signals. Texture analysis of ultrasound images is not recommended because it is easily affected by the system settings (e.g., built-in imaging processing, log-compression, or grayscale assignment), making the performance system-dependent. Comparatively, the analysis of raw image data (ultrasound backscattered signals) obtained without post-processing is a preferred solution to reduce the dependency of system characteristics. Based on the randomness of ultrasound backscattering, statistical distributions can be employed to model backscattered statistics (i.e., the echo amplitude distribution). This facilitates the analysis of image speckle patterns and characterization of the microstructures of the liver parenchyma [5], which is essentially a medium consisting of numerous acoustic scatterers (hepatocytes) [6]. The homodyned-K (HK) and Nakagami distribution models in particular provide a generalized description of ultrasound backscattered statistics measured from biological tissues [7]. Ultrasound parametric imaging based on the HK [8,9] and Nakagami models [10,11] has been shown to support the identification of hepatic steatosis. However, a prerequisite for using both methods is that ultrasound envelope data conform to the used distribution [12]. Therefore, Shannon entropy from information theory, a measure of signal uncertainty or complexity [12,13], has been proposed as a more flexible solution for parametric imaging of backscattered statistics without considering the statistical properties of ultrasound data [14]. Ultrasound entropy imaging has improved grading for hepatic steatosis and may be a steatosis-sensitive imaging biomarker [14,15,16,17].

Utilizing backscattered statistics analysis based on information entropy to characterize hepatic steatosis means that researchers must understand the fundamentals of acoustics and observe image features. By comparison, deep learning techniques provide an opportunity to automatically develop useful features for classification [18]. Convolutional neural networks (CNNs) have been successfully applied to medical image and pattern recognition, although a large amount of input data is required for successful training [19]. Obtaining comprehensively annotated datasets in the medical imaging domain remains a challenge. Against this background, transfer learning (i.e., fine-tuning CNN models pretrained on natural image datasets such as ImageNet) is an effective solution [20], and its use has been reported in ultrasound image analysis tasks [19,21,22]. Assessing the applicability of each pretrained model beforehand is difficult because it depends on the nature of the problems to be solved. Landmark studies have tended to use a version of GoogleNet called Inception v3; however, AlexNet or other simple models such as VGG are popular in the analysis of medical data [23]. Indeed, VGG-16 has been reported to outperform Inception v3 in classifying medical images [24,25]. It has also accurately identified speckle patterns for the classification of hepatic steatosis [26].

To date, no research has clarified and compared the clinical roles and value of entropy-based backscattered statistical analysis and pretrained models in the assessment of hepatic steatosis. Therefore, we explored the diagnostic performances of ultrasound entropy imaging and the VGG-16 model in grading hepatic steatosis using a pathologically proven dataset. In the following sections, we describe the experimental design, including data acquisition, grouping, processing, network architecture, algorithms for parametric imaging, and statistical analysis. The primary results indicated that ultrasound entropy imaging and VGG-16 can grade hepatic steatosis; however, ultrasound entropy imaging outperformed VGG-16 in diagnosing moderate or severe hepatic steatosis. Compared with deep learning, ultrasound entropy imaging better simultaneously satisfied both grading and clinical interpretations of hepatic steatosis.

## 2. Materials and Methods

### 2.1. Participants

All subjects gave their informed consent for inclusion before they participated in the study. The study was conducted in accordance with the Declaration of Helsinki and was approved by the Ethics Committee of Chang Gung Memorial Hospital in Taiwan (Approval No.: 201601928B0C501) to reuse clinical data obtained between 2017 and 2020. A total of 205 patients with confirmed chronic hepatitis B or C infection who were evaluated by gastroenterologists to need further liver biopsy examinations or partial liver resection were enrolled, and those with a history of liver resection, medications, alcohol abuse, or focal hepatic steatosis were excluded from the data analysis. Blood tests and ultrasound measurements for each patient were conducted after 8 h of overnight fasting. Liver resection or percutaneous liver biopsy was then performed within 1 week for histological examinations to grade hepatic steatosis. The demographic information, blood test results, and histological data are presented in Table 1. Based on the histological findings, hepatic steatosis was graded as normal (steatosis involving <5% hepatocytes), mild (5–33%), moderate (33–66%), or severe (>66%) [27].

### 2.2. Ultrasound Protocol

Abdominal ultrasonography of each participant was performed using a clinical ultrasound system (Model 3000, Terason, Burlington, MA, USA). A convex array transducer (Model 5C2A, Terason) with a pulse length (PL) of 2.3 mm and a central frequency of 3.5 MHz was employed. The liver parenchyma was imaged using intercostal scanning [28] by an experienced radiologist who did not have access to the patients’ medical data. Five independent scans were performed to obtain raw image radiofrequency (RF) data (focus: 4 cm; depth: 8 cm; width: 12.4 cm; line density: 256 scan lines of backscattered signals; each scan line had 1247 sampling points; sampling rate: 30 MHz). The digital gain index of the Terason system was set to 4, ensuring that the signal-to-noise ratio (SNR) of the raw image data was >20 dB [29].

### 2.3. Ultrasound Data Preprocessing

**Figure 1:**
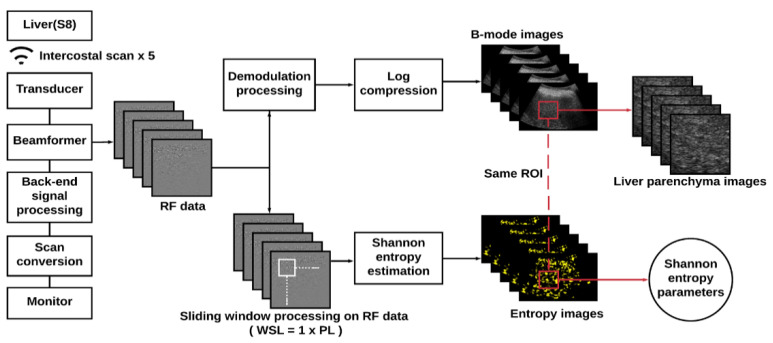
Algorithmic scheme for ultrasound data preprocessing and parametric imaging. An ultrasound envelope image obtained from the absolute value of the Hilbert transform of backscattered signals was used for B-mode imaging; raw image radiofrequency (RF) data were used for entropy imaging. A region of interest (ROI) with a fixed size (3.5 × 3.5 cm^2^) was used to obtain subimages corresponding to the liver parenchyma (WSL: window side length; PL: pulse length).

Refer to Figure 1. For each raw image, demodulation was carried out by calculating the absolute values of the Hilbert transform of each backscattered signal to obtain the envelope image, which was processed using logarithmic transform and scan conversion for B-mode imaging (700 pixels in width and 450 pixels in height) at a dynamic range of 40 dB. The raw image RF data were processed using a sliding window technique to construct ultrasound entropy parametric images in accordance with the algorithms described subsequently. First, a square window was set up in the upper-left corner of the image RF data to collect local backscattered signals. This was normalized in amplitude to construct a statistical histogram from which the probability density function and the corresponding entropy value were estimated using

(1) $$H_{C}=-\overset{n}{\underset{i=1}{\sum }}w\left(y_{i}\right)\log_{2}\left[w\left(y_{i}\right)\right]$$

where *y_i_* is the discrete random variable of the backscattered signals, *w*(*y_i_*) represents the probability value, and *n* indicates the number of bins. The estimated entropy value was used as a new pixel in the window location. The window then slid throughout the entire RF data to calculate local entropy values that were used to generate a parametric map, which was in turn resized using two-dimensional linear interpolation to have the same data size as the raw RF data. Pseudocolor was employed to display the entropy map, which was superimposed onto the B-mode image to reveal anatomical information and backscattered statistics. Regarding computational settings, 100 bins are needed to generate a histogram [15]. The window side length was set to one PL to implement small-window entropy imaging and improve the resolution [16,30]. The overlap ratio when the window was sliding was set to 50% to ensure a balance between image smoothness and computational efficiency [30]. For each B-mode image, manual segmentation was performed by an experienced physician on a region of interest (ROI) with a fixed size (3.5 × 3.5 cm^2^), which was applied to the corresponding entropy image to obtain subimages of the liver parenchyma. The B-mode and entropy subimages were then utilized for learning by the VGG-16 model and to estimate the average entropy values, respectively. 

### 2.4. Training and Evaluation

Each datum in the dataset was labeled according to different criteria for grading hepatic steatosis (Label 1: does not meet the criteria; Label 2: hepatic steatosis), as shown in Table 2. Based on the criterion used, the data were divided into training and test sets (the training-to-test ratio in the sample size was set to 4). To address the problem of class imbalance, data augmentation based on random cropping within the ROI was performed for the label with the fewest data to ensure that the amount of data for both labels in the training set was equal.

**Figure 2:**
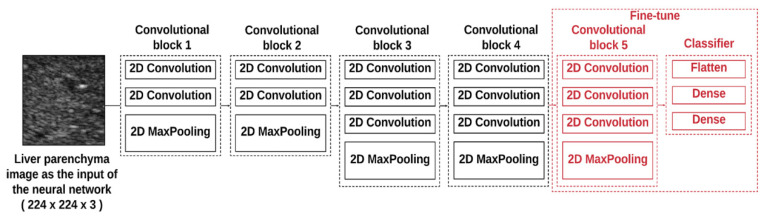
The architecture of the pretrained VGG-16 model was composed of five convolutional blocks (including the convolutional and maximum pooling layers). The input to the feature layer was a 224 × 224 square-pixel image of the liver parenchyma.

**Figure 3:**
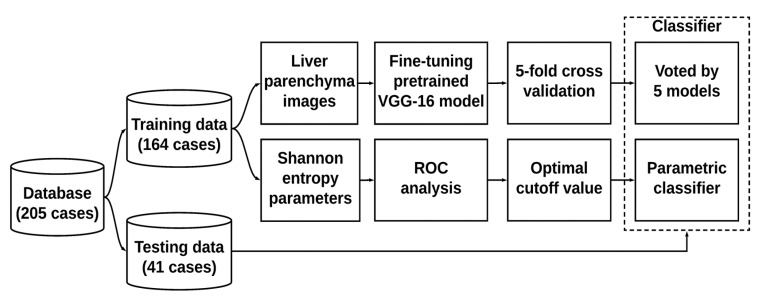
Data training and testing using VGG-16 and entropy imaging. The output of the VGG-16 model was determined by voting through five models from 5-fold cross-validation. The classification using ultrasound entropy imaging was determined in a comparison with the cutoff value obtained from receiver operating characteristic (ROC) analysis.

The architecture of the VGG-16 model pretrained on ImageNet was composed of five convolutional blocks (including the convolutional and maximum pooling layers), as shown in Figure 2. To reduce the computational complexity, the proposed VGG-16 model adopted a 3 × 3 convolution kernel with stride equal to 1 in the convolution layers and a 2 × 2 convolution kernel with stride equal to 2 in the maximum pooling layers. Three fully connected (FC) layers used for classification followed a stack of convolutional layers. The first two FC layers had 4096 nodes each, and the third FC layer utilized 1000 nodes to perform classification (one for each class). Rectified linear unit was used as the activation function for all hidden layers, and the softmax activation function was employed in the final FC layer. To increase the adaptability of the model for ultrasound imaging, fine-tuning was carried out whereby (i) the first four convolution blocks were frozen (using the pretrained weights). The final convolution block was optimized during training to preserve general features extracted in the previous layers and enhance the specificity of the model; (ii) the first two FC layers were modified to have 256 nodes each, and the output layer was adjusted to have 2 nodes to fulfill the binary classification for hepatic steatosis. Based on the architecture, 100 epochs and 5-fold cross-validation were used in the training phase by voting for the test dataset to determine the prediction. Concurrently, the average entropy values of each subject in the training dataset were utilized for receiver operating characteristic (ROC) curve analysis. The ROC curve was created by plotting the true positive rate against the false positive rate at various threshold settings. The cutoff entropy values for diagnosing different grades of hepatic steatosis were determined according to the point minimizing the Euclidean distance between the ROC curve and point (0, 1) [31] and used for testing purposes. The data training and tests performed using VGG-16 and entropy imaging are presented in Figure 3.

### 2.5. Statistical Analysis

To evaluate diagnostic performance, we calculated the sensitivity, specificity, accuracy, precision, recall, and F1-scores for hepatic steatosis grading in the test group. The ROC curves were also analyzed to obtain the areas under the ROC curves (AUROCs) with a 95% confidence interval (CI). The DeLong test was used to compare the AUROCs obtained using VGG-16 and ultrasound entropy imaging. Analyses were performed using MATLAB (R2019a, MathWorks, Natick, MA, USA) and SigmaPlot (version 12.0, Systat Software, Inc., San Jose, CA, USA). Statistical significance was set to *p* < 0.05.

## 3. Results

**Figure 4:**
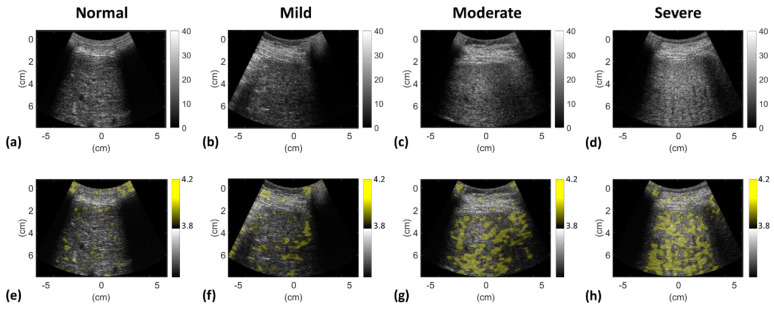
Typical ultrasound (**a**–**d**) B-mode and (**e**–**h**) entropy images obtained for different grades of hepatic steatosis. The brightness of the B-scan and entropy parametric images increased with the grade of hepatic steatosis, representing changes in the backscattered amplitude and statistics, respectively.

**Figure 5:**
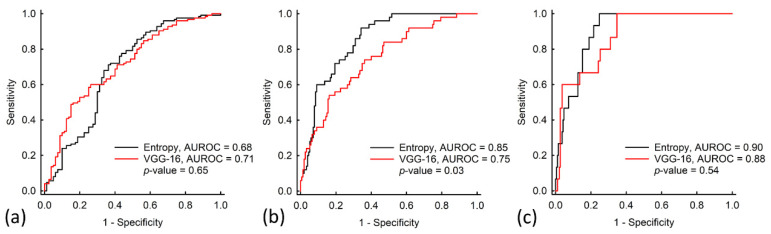
ROC curves when using the VGG-16 model and entropy estimation to grade hepatic steatosis as (**a**) ≥ mild (normal versus mild to severe), (**b**) ≥ moderate (normal to mild versus moderate to severe), or (**c**) ≥ severe (normal to moderate versus severe). The AUROCs for diagnosing hepatic steatosis from mild to severe grades when the VGG-16 model was used were 0.71, 0.75, and 0.88; those when entropy imaging was used were 0.68, 0.85, and 0.9. The AUROC when ultrasound entropy imaging was used to grade moderate hepatic steatosis was significantly higher than that when the VGG-16 model was used (*p* < 0.05).

Figure 4 depicts typical ultrasound B-mode and entropy images obtained for different grades of hepatic steatosis. The images suggest that the brightness of the B-scan and entropy parametric images increased with hepatic steatosis grade. Table 3 and Figure 5 present the cutoff entropy values, performance metrics, and AUROCs for hepatic steatosis diagnosis in the test group. These show that VGG-16 and entropy imaging can be used to assess hepatic steatosis. With respect to grading mild, moderate, and severe hepatic steatosis, the accuracies when VGG-16 was used were 70% (sensitivity: 73.18%; specificity: 60%), 80% (sensitivity: 63.25%; specificity: 74.82%), and 97% (sensitivity: 85.23%; specificity: 84.12%), respectively; those when ultrasound entropy imaging was used were 68% (sensitivity: 64.1%; specificity: 70.16%), 80% (sensitivity: 70%; specificity: 86.54%), and 83% (sensitivity: 78.82%; specificity: 93.3%), respectively. The AUROCs for grading hepatic steatosis from mild to severe when VGG-16 was used were 0.71, 0.75, and 0.88, respectively; those when entropy imaging was used were 0.68, 0.85, and 0.9. No significant differences in AUROCs were observed between VGG-16 and entropy imaging in grading mild and severe hepatic steatosis (*p* > 0.05). However, the AUROC for the use of ultrasound entropy imaging in grading moderate hepatic steatosis was significantly higher than that of VGG-16 (*p* < 0.05).

## 4. Discussion

### 4.1. The Significance of this Study

Regarding the characterization of hepatic steatosis, ultrasound entropy imaging and deep learning have different theoretical foundations and require different considerations in practice. The former involves physical descriptions and mathematical computations of backscattered signals to establish the link between the entropy and the grade of hepatic steatosis; the latter frequently uses pretrained CNNs for feature extraction and classification. By using the same clinical dataset, this study clarified the respective value of information entropy and deep learning in the ultrasonic detection of hepatic steatosis.

### 4.2. Comparisons of Information Entropy with Deep Learning

AUROC and accuracy were used in this study to evaluate learning algorithms; notably, they sometimes contradict each other, especially when the dataset is unbalanced [32]. For instance, the accuracy can be extremely high on a dataset for which numerous participants are in the same class. Therefore, the AUROC is recommended to replace accuracy in measuring and comparing classifiers because it is statistically consistent and more discriminating [32]. According to the above viewpoint, no significant difference was observed in diagnostic performance between VGG-16 and entropy imaging in detecting ≥mild and ≥severe hepatic steatosis; however, ultrasound entropy imaging outperformed the VGG-16 model in diagnosing ≥moderate hepatic steatosis. Interestingly, the use of a physics-based analysis method is as effective as using deep learning; it even provides improved performance in detecting moderate to severe hepatic steatosis.

The use of the pretrained VGG-16 model to detect hepatic steatosis was pioneered in a pilot study in which B-mode images (*n* = 157) were acquired for training, testing, and comparison with ultrasound diagnoses by experienced sonographers [26]. The diagnostic accuracy and AUROC in the binary classification of hepatic steatosis (normal vs. steatosis) were 90.6% and 0.96, respectively. In addition to the VGG-16, an Inception-ResNet-v2 model pretrained on ImageNet for classifying normal livers and steatosis (comprising ≥5% hepatocytes) was assessed for its effectiveness with patients with severe obesity undergoing wedge liver biopsy during bariatric surgery (*n* = 55) [19]. The accuracy was 0.96, and the AUROC was 0.97. The performance of a basic CNN architecture in grading hepatic steatosis was also assessed in a study involving 240 participants evaluated using sonographic findings. The results revealed a superior diagnostic ability in distinguishing moderate and severe hepatic steatosis (AUROC: 0.95) [33]. According to the above literature review, the diagnostic performance of VGG-16 in detecting hepatic steatosis does not appear to significantly differ from those of other deep learning models. Compared with the discussed studies, in the present study, we employed a relatively complete experimental design to incorporate both histological examinations and standard clinical grading criteria. However, discrepancies emerged in both the accuracies and AUROCs between the present work and previous studies. These discrepancies may be attributable to differences in the amount of data, diagnostic standards, and scanning approaches.

### 4.3. Considerations of Using Deep Learning in Grading Hepatic Steatosis

Deep learning should be used carefully in medical problems including the clinical detection of hepatic steatosis. This is because a fatty liver is significantly associated with nonviral hepatocellular carcinoma in patients; thus, strategies for liver cancer prevention, prediction, and surveillance require modification to involve considerations of hepatic steatosis [34]. Even if deep learning can predict hepatic steatosis, clinical decision-making regarding treatments and mechanisms for interpreting hepatic steatosis remains challenging due to the lack of medical physical information. This is also the reason why scientists suggest opening the black box of artificial intelligence to extend domain knowledge [35]. Nevertheless, this task remains challenging. Therefore, the use of complementary physics-based entropy analysis to characterize the liver is worth considering and of clinical merit, as supported by the experimental findings of this study.

### 4.4. Physical Interpretations of Information Entropy 

Hepatic steatosis typically includes microvesicular and macrovesicular steatosis [36]. In the condition of microvesicular steatosis, minute fat droplets uniformly distribute in the hepatocyte. Microvesicular steatosis is typically caused by acute liver failure; thus, it does not belong to a pathological feature of NAFLD [37]. In macrovesicular steatosis, however, a single large fat droplet, which is generally formed by the fusion of multiple small to medium-sized fat droplets [38], exists in the hepatocyte to push the nucleus to the periphery. Besides a single large fat droplet, small to medium-sized droplets in hepatocytes are also treated as features of macrovesicular steatosis [37], which is a major fatty change of NAFLD that equivalently increases the number of acoustic scatterers (fat droplets) in the scattering medium (liver parenchyma) [28].

In physical terms, Shannon entropy, based on information theory, is a quantitative measure of signal uncertainty and complexity. The entropy value estimated from ultrasound backscattered signals increases with fat infiltration in the human liver [14,15], indicating a promising ability to diagnose moderate and severe hepatic steatosis [16,17]. The entropy of ultrasound backscattered signals increases because the distribution density of scatterers (fat droplets) in the liver tissue increases [8], resulting in changes in the backscattered statistics from pre-Rayleigh to Rayleigh distributions [8,16]. Because advanced hepatic steatosis is a critical factor in grading nonalcoholic steatohepatitis and fatty liver disease [36], evaluating moderate and severe grades has clinical significance. Notably, the current results show that ultrasound entropy imaging was superior to VGG-16 in identifying moderate-to-severe hepatic steatosis. Thus, ultrasound entropy imaging can be used to simultaneously grade hepatic steatosis and explain the meaning of microstructure-related scatterings.

### 4.5. Limitations and Future Work

Some limitations in this study must be addressed. First, the diagnostic performances of VGG-16 and entropy imaging obtained after training were not ideal, the major cause of this probably being that the amount of data was insufficient. A larger amount of data is expected to improve the diagnostic performance in general. Thus, a large-scale clinical dataset is recommended for more reliable estimations of the accuracy and AUROC, as well as enhancement of the sensitivity and specificity. However, the above limitation does not affect comparisons of VGG-16 and entropy imaging based on the same dataset. Instead, with the limited amount of data used in this study, the superiority of ultrasound entropy imaging over VGG-16 in characterizing significant hepatic steatosis indicates the great potential of information entropy in future clinical applications. Second, ultrasound resolution is frequency-dependent, and the sensitivity of backscattered statistics-related parameters is dependent on transducer focusing. The effects of the beam profile on the diagnostic performances of deep learning and entropy imaging merit further exploration. Third, only one imaging system was used to collect data; however, the qualities of the image and backscattered signals (e.g., image contrast and signal-to-noise ratio) depend heavily on system characteristics. Therefore, a cross-platform investigation should be employed in future research. Fourth, considering that the information entropy of ultrasound backscattered signals conveys information associated with changes in microstructures, entropy imaging as the input for deep learning may be used as a new prospective strategy to provide extra clues to diagnose hepatic steatosis.

## 5. Conclusions

In summary, in this study, we compared the value and roles of deep learning and backscattering statistics analysis in the assessment of hepatic steatosis by applying the VGG-16 model and ultrasound entropy imaging to the same clinical dataset. The entropy measure outperformed VGG-16 in identifying participants with significant hepatic steatosis (moderate or severe). The novel insight offered by this study is that, compared with deep learning, ultrasound information entropy not only improved diagnosis but also conveyed microstructure-related physical information, which is clinically beneficial to interpretations of hepatic steatosis.

## Figures and Tables

**Table 1:** Demographic data of patients.

Characteristics	Value
**Male/Female**	130/75
**Age, years**	
Mean ± standard deviation (range)	55 ± 11.6 (24–79)
Median	57
**BMI, kg/m^2^**	
Mean ± standard deviation (range)	25.3 ± 3.8 (16.8–37.8)
Median	24.9
**AST, U/L**	
Mean ± standard deviation (range)	67.2 ± 68.6 (15–507)
Median	46
**ALT, U/L**	
Mean ± standard deviation (range)	86.8 ± 99.0 (8–595)
Median	52
**PLT, 10^3^/mm^3^**	
Mean ± standard deviation (range)	193 ± 68.2 (73–542)
Median	186
**Steatosis grade, no. of patients**	
Normal	79
Mild	74
Moderate	35
Severe	17

Note: Unless otherwise noted, data are numbers of patients. BMI: body mass index, PLT: platelet count, AST: aspartate aminotransferase, ALT: alanine aminotransferase. Normal AST levels for female and male participants are less than 35 U/L and 50 U/L, respectively. Normal ALT levels for female and male participants are less than 19 U/L and 30 U/L, respectively.

**Table 2:** Sample size and amount of data used for labeling, training, and tests to identify hepatic steatosis by using different criteria (Label 1: does not meet the criteria; Label 2: hepatic steatosis).

Group	1	2	3
Hepatic steatosis grade	≥mild	≥moderate	≥severe
(Number of subjects, Amount of data) for Label 1	(79, 395)	(153, 765)	(188, 940)
(Number of subjects, Amount of data) for Label 2	(126, 630)	(52, 260)	(17, 85)
(Number of subjects, Amount of data) for training set	(164, 820)	(164, 820)	(164, 820)
(Number of subjects, Amount of data) for test set	(41, 205)	(41, 205)	(41, 205)

**Table 3:** Performance metrics for diagnosing hepatic steatosis in the test group using ultrasound entropy imaging and a VGG-16 neural network (AUROC: the area under the receiver operating characteristic curve; CI: confidence interval).

Parameter	Shannon Entropy			VGG-16 Model		
	≥Mild	≥Moderate	≥Severe	≥Mild	≥Moderate	≥Severe
Cutoff value	3.75	3.79	3.82	N/A	N/A	N/A
Accuracy	0.68	0.80	0.83	0.70	0.80	0.97
Sensitivity, %	64.10	70.00	78.82	73.18	63.25	85.23
Specificity, %	70.16	86.54	93.30	60.00	74.82	84.12
Precision, %	58.62	93.86	99.33	54.12	88.39	97.93
Recall, %	63.75	69.03	78.42	73.75	63.87	74.73
F1-score	0.61	0.80	0.88	0.62	0.74	0.85
AUROC (95% CI)	0.68 (0.60–0.76)	0.85 (0.80–0.90)	0.90 (0.85–0.95)	0.71 (0.64–0.78)	0.75 (0.67–0.82)	0.88 (0.80–0.94)
DeLong test (*P*-value)	0.65	0.03	0.54	0.65	0.03	0.54
